# Assessing Functional Capacity in Directly and Remotely Monitored Home-Based Settings in Individuals With Chronic Respiratory Diseases: Protocol for a Multinational Validation Study

**DOI:** 10.2196/57404

**Published:** 2024-06-28

**Authors:** Alec Bass, Sarah Géphine, Mickaël Martin, Marianne Belley, Manon Robic, Claudine Fabre, Jean-Marie Grosbois, Geneviève Dion, Didier Saey, Arnaud Chambellan, François Maltais

**Affiliations:** 1 Centre de recherche de l’Institut universitaire de cardiologie et de pneumologie de Québec (CR-IUCPQ) Université Laval Québec, QC Canada; 2 FormAction Santé Pérenchies France; 3 Université of Lille, Université de Artois, Université du Littoral Cote d'Opale, ULR 7369 - URePSSS - Unité de Recherche Pluridisciplinaire Sport Santé Société Lille France; 4 Service de Pneumologie Hôpital Saint-Philibert Université Catholique de Lille Lomme France

**Keywords:** chronic obstructive pulmonary disease, COPD, exercise capacity test, interstitial lung diseases, physiotherapy, rehabilitation, telerehabilitation, validation study, stepper test

## Abstract

**Background:**

Pulmonary rehabilitation is widely recommended to improve functional status and as secondary and tertiary prevention in individuals with chronic pulmonary diseases. Unfortunately, access to timely and appropriate rehabilitation remains limited. To help close this inaccessibility gap, telerehabilitation has been proposed. However, exercise testing is necessary for effective and safe exercise prescription. Current gold-standard tests, such as maximal cardiopulmonary exercise testing (CPET) and the 6-minute walk test (6MWT), are poorly adapted to home-based or telerehabilitation settings. This was an obstacle to the continuity of services during the COVID-19 pandemic. It is essential to validate tests adapted to these new realities, such as the 6-minute stepper test (6MST). This test, strongly inspired by 6MWT, consists of taking as many steps as possible on a “stepper” for 6 minutes.

**Objective:**

This study aims to evaluate the metrological qualities of 6MST by (1) establishing concurrent validity and agreement between the 6MST and CPET, as well as with the 6MWT; (2) determining test-retest reliability in a home-based setting with direct and remote (videoconferencing) monitoring; and (3) documenting adverse events and participant perspectives when performing the 6MST in home-based settings.

**Methods:**

Three centers (Centre de recherche de l’Institut universitaire de cardiologie et de pneumologie de Québec in Québec, Groupement des Hôpitaux de l’Institut Catholique de Lille in France, and FormAction Santé in France) will be involved in this multinational project, which is divided into 2 studies. For study 1 (objective 1), 30 participants (Québec, n=15; France, n=15) will be recruited. Two laboratory visits will be performed to assess anthropometric data, pulmonary function, and the 3 exercise tolerance tests (CPET, 6MWT, and 6MST). Concurrent validity (paired sample t tests and Pearson correlations) and agreement (Bland-Altman plots with 95% agreement limits) will be evaluated. For study 2 (objectives 2 and 3), 52 participants (Québec, n=26; France, n=26) will be recruited. Following a familiarization trial (trial 1), the 6MST will be conducted on 2 separate occasions (trials 2 and 3), once under direct supervision and once under remote supervision, in a randomized order. Paired sample t test, Bland-Altman plots, and intraclass correlations will be used to compare trials 2 and 3. A semistructured interview will be conducted after the third trial to collect participants’ perspectives.

**Results:**

Ethical approval was received for this project (October 12, 2023, in Québec and September 25, 2023, in France) and the first participant was recruited in February 2024.

**Conclusions:**

This study innovates by validating a new clinical test necessary for the development and implementation of new models of rehabilitation adapted to home and telerehabilitation contexts. This study also aligns with the United Nations Sustainable Development Goals by contributing to augmenting health care service delivery (goal 3) and reducing health care access inequalities (goal 11).

**Trial Registration:**

ClinicalTrials.gov NCT06447831; https://clinicaltrials.gov/study/NCT06447831

**International Registered Report Identifier (IRRID):**

DERR1-10.2196/57404

## Introduction

### Background

Recent guidelines strongly recommend pulmonary rehabilitation for individuals with stable or acutely exacerbated chronic obstructive pulmonary disease (COPD) or with interstitial lung disease (ILD) [1]. Unfortunately, access to rehabilitation care remains limited in many countries, including Canada and France. Indeed, the latest available estimates indicate that only 0.4% of individuals in Canada and 8.6% of individuals in France with COPD have access to pulmonary rehabilitation programs [2,3]. The development of new and innovative health care models, including in rehabilitation, is urgently needed to address this health care gap that is only expected to worsen with the aging populations in most advanced economies [2,4]. Home-based rehabilitation and telerehabilitation are now increasingly considered as a solution to increase rehabilitation access in this population [1,4,5]. These programs are cost-effective alternatives to traditional ambulatory pulmonary rehabilitation models anchored in specialized hospital institutions [6,7]. Moreover, the effectiveness of home-based rehabilitation and pulmonary telerehabilitation programs has been demonstrated and is comparable to traditional rehabilitation models in individuals with COPD [8]. Yet, clinicians report the lack of validated functional exercise capacity tests for these new models of care as a major barrier for their implementation [9,10]. These tests are important to ensure safe, effective, and personalized exercise prescription during rehabilitation, and to monitor and document its effects [11,12].

The current gold standard for exercise capacity testing is maximal cardiopulmonary exercise testing (CPET), which is generally conducted on a cycle ergometer or a treadmill. These tests are designed to assess maximal physiological responses to exercise and thus require expensive equipment (eg, gas exchange analyzer and electrocardiogram) that are not easily accessible outside of specialized institutions. Moreover, these tests are not always representative of functional activities in daily life (eg, cycling may not be an activity of daily life for many individuals) [13]. Thus, functional capacity tests have been widely adopted in ambulatory rehabilitation settings [13,14]. To this effect, the 6-minute walk test (6MWT), which only requires an unobstructed corridor (≥30 meters), is among the most widely used [13,15]. Despite its simplicity, the 6MWT is not adapted to the realities of home-based and telerehabilitation settings. Indeed, limited available space inhibits its realization and has been highlighted as a barrier to the continuity of rehabilitation services during the COVID-19 pandemic [9]. It is therefore essential to validate functional capacity tests adapted to this new reality, such as the 6-minute stepper test (6MST). This test, strongly inspired by 6MWT, consists of taking as many steps as possible on a “stepper” for 6 minutes [16,17].

In individuals with COPD, initial studies indicate that the 6MST is reliable, as no significant difference in test results was found when repeated on consecutive days in a laboratory setting [16]. The 6MST is also valid, as the number of steps taken during the test is correlated with the total distance traveled during the 6MWT (*r*=0.56-0.87, *P*<.01), peak power during CPET (*r*=0.46, *P*<.01), and peak oxygen consumption during CPET (*r*=0.39, *P*<.01) [17,18]. Responsiveness to change following pulmonary rehabilitation has also been shown, with a minimally important difference estimated at 40 steps [19]. Finally, predictive equations have been computed to individualize aerobic training intensity using the 6MST [20]. However, to our knowledge, cardiorespiratory responses during the 6MST in individuals with COPD have not been formally compared with those during the 6MWT and CPET to date. This is particularly important when considering the development of home-based and telerehabilitation programs, as different cardiorespiratory responses to testing may elicit safety concerns. In individuals with ILD, there is limited evidence regarding the validity of the 6MST, as the number of steps taken during the test is correlated with the total distance traveled during the 6MWT (*r*=0.70, *P*<.01) [21]. Interestingly, in individuals with ILD, cardiorespiratory responses during the 6MST have been compared with the 6MWT. In this population, the 6MST leads to greater heart rate and ventilatory responses, and blood oxygen saturation remains higher during the 6MST [21,22]. Finally, as the 6MST has only been validated in laboratory or hospital settings, metrological properties in home-based settings with direct or remote (ie, videoconferencing) monitoring are currently unknown in individuals with COPD or ILD.

### Objectives and Hypotheses

#### Overview

The overall aim of this project is to provide further evidence regarding the metrological properties of the 6MST in individuals with COPD or ILD. Specific objectives and hypotheses are described below.

#### Objective 1

Objective 1 is to determine concurrent validity and the level of agreement (or bias) between peak cardiorespiratory responses and reported symptomology during the 6MST and CPET and the 6MST and 6MWT. The corresponding hypotheses are that peak cardiorespiratory responses and reported symptomology to the 6MST will be strongly correlated to those measured during CPET and the 6MWT (ie, concurrent validity), and that these values will be systematically lower than those measured during CPET (ie, presence of bias), but similar to those measured during the 6MWT (ie, no bias).

#### Objective 2

Objective 2 is to determine test-retest reliability for the 6MST in home-based settings under direct and remote (ie, videoconferencing) monitoring. The corresponding hypothesis is that measurement error will be minimal and intraclass correlations will be good and similar between direct and remote supervision.

#### Objective 3

Objective 3 is to determine the safety and the perception of the 6MST in individuals with chronic pulmonary diseases in home-based settings under direct and remote monitoring by collecting adverse events and qualitative feedback. The corresponding hypothesis is that the test will be safe (ie, absence of serious adverse events) and individuals will report a good level of overall satisfaction with the test, irrespective of the type of supervision.

## Methods

### Research Design

This research project comprises 2 studies resulting from an international collaboration between the Centre de recherche de l’Institut universitaire de cardiologie et de pneumologie de Québec (CR-IUCPQ, Université Laval, Canada), the Groupement des Hôpitaux de l’Institut Catholique de Lille (GHICL, France), and FormAction Santé (France). Study 1 (objective 1) is a prospective, multicentric, quantitative, and validation study realized in an outpatient hospital setting at the CR-IUCPQ and the GHICL. Study 2 (objectives 2 and 3) is a prospective, multicentric, mixed methods, and validation study realized at the participants’ homes in Québec (CR-IUCPQ) and in northern France (FormAction Santé).

### Participants and Inclusion and Exclusion Criteria

A quota sampling method, based on disease and sex (ie, 1:1), will be used. All sample sizes were calculated a priori with an open-source calculator available on the internet or SPSS (IBM) [23]. Inclusion and exclusion criteria are summarized in Textbox 1.

**Inclusion and exclusion criteria.**

**Inclusion criteria**

- Adults ≥40 years old
- Diagnosis of chronic obstructive pulmonary disease (Global Initiative for Chronic Obstructive Lung Disease Diagnostic Criteria 2 to 4) or of interstitial lung disease (>6 months)
- Clinically stable for ≥4 weeks

**Exclusion criteria**

- Interstitial lung disease associated with connective tissue disease and sarcoidosis (often accompanied by multisystemic effects)
- Unstable or severe cardiac condition
- Invalidating rheumatologic or neurologic condition
- Weight exceeding maximal limits of exercise equipment (eg, 130 kg for the stepper)
- Any other physical or medical condition limiting or contraindicating exercise testing
- Simultaneous participation in another study requiring changes in medication
- Recent pulmonary rehabilitation (≤1 year, potential learning bias for the 6-minute walk test and the 6-minute stepper test)
- For study 1 only: use of oxygen therapy (unable to provide oxygen therapy during testing due to limitation of gas exchange analysis equipment)
- For study 2 only: participation in study 1

For study 1, a mean difference of 0.28±0.32 L/min in maximal oxygen uptake between the 6MST and CPET was assumed, based on previous studies between the 6MWT and CPET [22,24]. To detect a difference of this amplitude with a paired sample *t* test, assuming a power of 80%, an α level of .05, and a dropout rate of 15%, 15 participants are needed. Fifteen individuals of each disease population (ie, COPD, n=15; ILD, n=15) will be included for a total sample of 30 participants (Québec, n=15; France, n=15), as cardiorespiratory responses to exercise are different among these populations [25].

For study 2, a minimal acceptable intraclass correlation coefficient of 0.5, and an expected intraclass correlation coefficient of 0.75, were assumed for the test-retest reliability of the 6MST. Assuming a power of 80%, an α level of .05, and a dropout rate of 15%, 52 participants (Québec, n=26; France; n=26) will be recruited. This calculated sample was taken as a whole, as test-retest reliability is not expected to be influenced by the type of respiratory disease.

### Study 1: Procedures and Measurements

For study 1, in addition to a review of medical records, two 3-hour laboratory visits (separated by 6-18 days) will be conducted. Participants will be instructed to take all medications as usual, including inhalers. A summary of collected data and outcomes is presented in Table 1.

**Table 1:** Data collected during study 1.

Measurements		Medical records	Visit 1		Visit 2
**Sociodemographic**					
	Age	✓			
	Sex	✓			
	Pulmonary diagnosis	✓			
	Comorbidities and risk factors	✓			
**Anthropometric**					
	Mass (kg)		✓		
	Height (m)		✓		
	Composition (bioimpedance)		✓		
	Leg length (cm)		✓		
**Pulmonary function**					
	Spirometry		✓		
	Plethysmography		✓		
	DLCO^a^		✓		
**CPET^b^ on cycle ergometer**					
	Dyspnea (/10)^c^		✓		
	Leg fatigue (/10)^c^		✓		
	Absolute oxygen uptake (L/min)		✓		
	Relative oxygen uptake (mL/kg·min)		✓		
	Carbon dioxide excretion (L/min)		✓		
	Minute ventilation (L/min)		✓		
	Tidal volume (L)		✓		
	Respiratory rate (breath/min)		✓		
	Heart rate (bpm)		✓		
	Blood oxygen saturation (%)		✓		
	Blood pressure (mm Hg)		✓		
	Québec only: inspiratory capacity (pre and post)		✓		
**6MWT^a^**					
	Total distance traveled (m)			✓	
	Identical measurements as CPET			✓	
**6MST^e^**					
	Total number of steps			✓	
	Identical measurements as CPET			✓	

^a^DLCO: diffusing capacity of the lungs for carbon monoxide.

^b^CPET: maximal cardiopulmonary exercise testing.

^c^/10: modified Borg Scale rating.

^d^6MWT: 6-minute walk test.

^e^6MST: 6-minute stepper test.

#### Review of Medical Records

Medical records will be reviewed, and the information retrieved regarding participant sociodemographic characteristics will be age, sex, pulmonary diagnosis, medications, comorbidities, and cardiovascular risk factors. Physical activity level will be assessed using the Ricci and Gagnon questionnaire [26].

#### First Laboratory Visit

During the first laboratory visit, anthropometric measurements, pulmonary function tests, and CPET on a cycle ergometer will be performed.

#### Anthropometric Measurements

Anthropometric measurements will be composed of body mass, height, body composition (ie, lean and fat mass), and leg length. A standardized medical scale and stadiometer will be used to measure body mass and height, respectively. Body composition will be estimated by bioimpedance [27]. Leg length will be measured using a standard tape measure from the anterior superior iliac spine to the medial malleoli [28,29]. The mean of two measurements of the right leg will be used [28,29].

#### Pulmonary Function

Pulmonary function will be characterized by spirometry, lung volume assessment (plethysmography), and diffusing capacity of the lungs for carbon monoxide, following current guidelines [30-32]. Spirometry will be conducted first [30]. After a period of quiet breathing, participants will be instructed to inhale to total lung capacity and exhale quickly and forcefully to residual volume. Forced vital capacity and forced expiratory volume in 1 second will be recorded. The test will be repeated until an agreement of at least 5% is obtained for 3 measures of forced vital capacity. The mean values of these 3 tests will be recorded (forced vital capacity and forced expiratory volume in 1 second) [30]. Plethysmography will be conducted second [31]. Participants will be instructed to breathe quietly, hands on their cheeks, until end-expiratory lung volume is stable. The shutter will be closed near functional residual capacity, and participants will be instructed to conduct a series of gentle pants (between 0.5 and 1 Hz) in order to obtain at least 3 with adequate technique. The shutter will then be opened, and the participant will exhale to residual volume, followed by an inspiratory slow vital capacity maneuver. The test will be repeated until an agreement of at least 5% is obtained for 3 measures of functional residual capacity. The mean value of these 3 tests will be recorded (total lung capacity, slow vital capacity, inspiratory capacity, functional residual capacity, expiratory reserve volume, and residual volume) [31]. The diffusing capacity of the lungs for carbon monoxide will be measured third [32]. After a period of relaxed tidal breathing, participants will exhale slowly to residual volume, followed by a rapid inhalation to total lung capacity during which a standardized gas mixture will be provided (carbon monoxide, methane, oxygen, and nitrogen). Participants will be instructed to hold their breath for 8 to 12 seconds before exhaling to residual volume. The test will be repeated following a 4-minute rest period. At least 2 acceptable tests will be obtained [32].

#### Maximal Cardiopulmonary Exercise Testing

CPET will be performed on a cycle ergometer (Quinton Corival 400 in Québec and Ergoline Ergoselect 200 in France) in accordance with the current recommendations [33]. The protocol used is presented in Textbox 2. Briefly, following a warm-up, the work rate will increase by 10 to 15 watts per minute, following a ramp protocol, until the criteria for maximality is reached. The criteria for maximality will be those recommended by the American College of Sports Medicine and include abnormal cardiovascular responses or signs of ischemia, signs or symptoms of cerebral hypoxemia, marked pulsed blood oxygen desaturation (SpO_2_≤80%), and at the request of the participant [34]. Participants will be asked to maintain a pedaling rate of 60±5 revolutions per minute. The modified Borg scale (/10) will be used to assess dyspnea and leg fatigue at rest before the test, during (every 2 minutes and at peak exercise), and after the test (5 minutes following the end of the test). Cardiorespiratory responses during the CPET will be monitored continuously using a breath-by-breath gas analyzer (Oxycon Mobile in Québec and Metamax 3B Cortex in France), an electrocardiogram, and an oximeter (Nonin). This equipment will provide data for absolute and relative oxygen uptake, carbon dioxide excretion, minute ventilation, tidal volume, respiratory rate, heart rhythm and rate, and pulsed oxygen saturation (SpO_2_). Blood pressure will be measured manually before and after the test. In Québec only, inspiratory capacity will be measured before and within 10 seconds of the end of the test (sitting on the cycle ergometer and hands gripping the handlebars) and a reduction of more than 150 mL will confirm the occurrence of dynamic hyperinflation [35].

**Protocol for CPET on the stationary cycle ergometer.**

**Resting phase**

- Duration: ≥3 minutes (following equipment set-up)
- Resting measurements recorded during the last minute of this phase

**Warm-up phase**

- Duration: 3 minutes following resting phase
- Warm-up at an equivalent of 20 watts and at a pedaling rate of 60±5 revolutions per minute

**Incremental phase**

- Duration: 8 to 12 minutes
- Work rate: start at 20 watts and increase by 10 to 15 watts per minute (ramp)
- Pedaling rate: 60±5 revolutions per minute (stop test if individual falls under 50 revolutions per minute)

**Recovery phase**

- Total duration: 5 minutes
- Active phase (pedaling at 20 watts) for 2 minutes, followed by passive phase (quiet sitting) for 3 minutes

**Monitoring**

- Continuous: breath-by-breath gas analysis, electrocardiography, peripheral oxygen saturation (stop test if pulsed oxygen saturation ≤80% with symptoms, such as dizziness, nausea, etc)
- During the last minute of the unloaded phase, every 2 minutes during the incremental phase, immediately at completion, and at 5 minutes of the recovery phase: blood pressure, dyspnea (/10), and leg fatigue (/10)
- Pre- and postincremental phase (Québec only): inspiratory capacity

To address current international recommendations that require the test to be repeated twice, the first laboratory visit will end with a familiarization trial of the 6MWT for individuals who have not done the test during the previous 12 months (the 6MWT is routinely conducted during medical follow-ups in the centers in this study) [36,37]. Data from this familiarization trial will not be recorded (although vital signs will be monitored for safety).

#### Second Laboratory Visit

During the second laboratory visit, spirometry will be repeated to ensure the stability of the participants’ health state (particularly relevant for individuals with COPD). Thereafter, the 6MST and 6MWT will be performed in a randomized order, separated by a rest period of at least 30 minutes.

The 6MWT will be performed in a leveled, unobstructed corridor in accordance with current recommendations [36,37]. Briefly, the 30-meter testing area will be marked at both extremities with brightly colored cones. Standardized instructions will be given before beginning the test, and standardized encouragements will be provided every minute during (see Textbox 3 for instructions in French used in this study) [36]. The total distance traveled will be collected to the nearest meter. Dyspnea, leg fatigue, and cardiorespiratory responses will be collected as described previously.

**Standardized instructions and encouragements during the 6-minute walk test. See Multimedia Appendix 2 for the English version.**

**Standardized instructions in French**

Le but de ce test est de marcher la plus grande distance possible pendant 6 minutes. Vous devrez réaliser des allers-retours dans ce couloir. Six minutes, c’est une longue durée, vous risquez de vous essouffler et de vous fatiguer. Vous pouvez choisir de ralentir ou d’arrêter, si nécessaire. Vous pouvez vous appuyer contre le mur pour vous reposer, mais recommencer la marche dès que possible.

Vous ferez des allers-retours en contournant les cônes orange. Vous devriez faire des virages rapides afin de poursuivre dans la direction opposée sans hésitation. Je vais vous montrer comment faire le virage, regardez-moi. (Démonstration)

Êtes-vous prêts à faire le test? Je vais compter le nombre d’aller-retour que vous faites et je vous dirai la durée restante toutes les minutes. Rappelez-vous, l’objectif du test est de marcher la plus grande distance possible pendant 6 minutes, mais vous ne devez pas courir. Je vais compter à trois et dire « GO ». Partez lorsque je dis « GO ».

**Standardized encouragements in French**

- C’est bien. Il vous reste 5 minutes.
- Continuez comme ça. Il vous reste 4 minutes.
- C’est bien. Vous avez fait la moitié.
- Continuez comme ça. Il vous reste seulement 2 minutes.
- C’est bien. Il vous reste seulement 1 minute.

The 6MST will be performed in accordance with the original description [16]. The test consists of taking the maximum number of steps on a stepper in 6 minutes. One step is counted for each complete flexion-to-extension movement of a lower extremity. The stepper used will be standardized and identical for all participants (Zinnor Mini Multifunctional Fitness Stepper [Zinnor] in Québec and Athletic Go Sport in France). Participants will be able to lightly touch a stable surface (ie, a wall) for balance, if needed. Participants will be allotted a 2-minute familiarization period, followed by rest (≥3 minutes seated), then will begin the test. Standardized validated instructions and encouragements, adapted from those of the 6MWT, will be provided (Textbox 4) [16,38]. Evaluators will ensure that complete lower extremity extension is reached during every step. The total number of steps will be automatically provided by the stepper. Dyspnea, leg fatigue, and cardiorespiratory responses will be collected as described previously.

**Standardized instructions and encouragements during the 6-minute stepper test. See Multimedia Appendix 3 for the English version.**

**Standardized instructions in French**

Le but de ce test est de réaliser le plus grand nombre de pas possible pendant 6 minutes. Six minutes, c’est une longue durée, vous risquez de vous essouffler et de vous fatiguer. Vous pouvez choisir de ralentir ou d’arrêter, si nécessaire. Vous pouvez vous appuyer contre le mur pour vous reposer, mais recommencer l’exercice dès que possible.

Vous devez étendre complètement la jambe pour réaliser chaque pas. Le step doit toucher la base du stepper à chaque pas. Je vais vous montrer le bon mouvement, regardez-moi. (Démonstration)

Êtes-vous prêts à faire le test? L’appareil va compter le nombre de pas que vous faites et je vous dirai la durée restante toutes les minutes. Rappelez-vous, l’objectif du test est de réaliser le plus grand nombre de pas pendant 6 minutes. Je vais compter à trois et dire « GO ». Partez lorsque je dis « GO ».

**Standardized encouragements in French**

- C’est bien. Il vous reste 5 minutes.
- Continuez comme ça. Il vous reste 4 minutes.
- C’est bien. Vous avez fait la moitié.
- Continuez comme ça. Il vous reste seulement 2 minutes.
- C’est bien. Il vous reste seulement 1 minute.

### Study 2: Procedures and Measurements

For study 2, in addition to a review of medical records, three 1-hour home visits (separated by 2-5 days) will be conducted. Participants will be instructed to take all medications as usual, including inhalers. Individuals who participated in study 1 will not be admissible for study 2 to reduce the risk of bias introduced by a possible learning effect with the 6MST. A summary of collected data and outcomes is presented in Table 2 and further described below.

**Table 2:** Data collected during study 2.

Measurements			Medical records	Trial 1	Trial 2		Trial 3
**Sociodemographic**							
	Age	✓					
	Sex	✓					
	Pulmonary diagnosis	✓					
	Comorbidities and risk factors	✓					
**Anthropometric**							
	Mass (kg)	✓					
	Height (m)	✓					
**General lower limb function**							
	SPPB^a^ (/12)			✓			
**6MST^b^**							
	Total number of steps			✓	✓	✓	
	Dyspnea (/10)^c^			✓	✓	✓	
	Leg fatigue (/10)^c^			✓	✓	✓	
	Heart rate (bpm)			✓	✓	✓	
	Pulsed oxygen saturation (%)			✓	✓	✓	
**Adverse events**							
	Minor			✓	✓	✓	
	Serious			✓	✓	✓	
**Semistructured interview**							
	Level of comfort during test					✓	
	Perception of safety					✓	

^a^SPPB: Short Physical Performance Battery.

^b^6MST: 6-minute stepper test.

^c^/10: modified Borg Scale rating.

#### Review of Medical Records

Medical records will be reviewed, and the following information will be retrieved regarding participant sociodemographic characteristics: age, sex, pulmonary diagnosis, medications, comorbidities, and cardiovascular risk factors. Physical activity level will be assessed using the Ricci and Gagnon questionnaire [26].

#### First Trial

The first trial of the 6MST (familiarization trial) will be conducted at the participant’s home with the therapist present. Preceding this trial, the Short Physical Performance Battery will be performed to obtain a general assessment of lower limb function [39].

The 6MST will be performed using a standardized portable stepper (same as in study 1) and following the same protocol as in study 1, including the 2-minute pretest warm-up period. However, full cardiorespiratory responses will not be measured. Instead, an oximeter (Nonin) will be used to monitor heart rate and SpO_2_ continuously. Blood pressure, dyspnea, and perceived exertion will be measured pre*-* and posttest using the modified Borg scale (/10). For individuals on home oxygen therapy, oxygen flows will be titrated if SpO_2_ falls below 88% during the test, with the aim of maintaining SpO_2_ between 88% and 92%, while always respecting the flow limits of the prescribing physician’s parameters. As in study 1, the test will be terminated if blood oxygen saturation falls to ≤80% accompanied by symptoms of hypoxia (such as dizziness, nausea, etc).

All adverse events will be recorded and graded according to the Common Terminology Criteria for Adverse Events, version 5 (United States Department of Health and Human Services), progressing from mild (1) to lethal (5) [40]. Textbox 5 presents the standardized grading used in this study. Adverse events are defined as any event that leads to (or could potentially lead to) prejudice to the participant or that inhibits the completion of the test. Examples include dyspnea, dizziness or nausea, close-call or fall events, musculoskeletal pain, chest pain, syncope, etc.

**Classification of adverse events based on the Common Terminology Criteria for Adverse Events, version 5.**

**Grade 1: Mild**

Mild adverse events are asymptomatic or with mild symptoms, or do not require intervention. Such adverse events include

- Musculoskeletal pain or injury (asymptomatic or mild)
- Joint effusion (asymptomatic or mild)
- Dyspnea with moderate exertion
- Productive cough with minimal production of sputum
- Wheezing with minimal symptoms
- Dehydration requiring increased oral fluid intake
- Hypoglycemia or hyperglycemia not requiring medical intervention
- Asymptomatic hypotension

**Grade 2: Moderate**

Moderate adverse events require minimal, local, or noninvasive intervention, or limit age-appropriate instrumental activities of daily living (preparing meals, doing the groceries, managing money, etc). Such adverse events include

- Musculoskeletal pain or injury limiting instrumental activities of daily living
- Joint effusion limiting instrumental activities of daily living
- Dyspnea with minimal exertion limiting instrumental activities of daily living
- Productive cough with moderate production of sputum or limiting instrumental activities of daily living
- Wheezing with moderate symptoms, or requiring medical intervention, or limiting instrumental activities of daily living
- Dehydration requiring intravenous fluids
- Hypoglycemia or hyperglycemia requiring intervention but no long-term changes in medical management
- Hypotension requiring nonurgent intervention
- Chest pain of palpitations related to exertion subsiding with rest

**Grade 3: Severe**

Severe adverse effects are medically significant but not immediately life-threatening, or require hospitalization, or limit self-care activities of daily living (bathing, dressing, using the toilet, feeding self, etc). Such adverse events include

- Musculoskeletal pain or injury limiting self-care activities of daily living
- Joint effusion limiting self-care activities of daily living
- Dyspnea at rest limiting self-care activities of daily living
- Productive cough with copious production of sputum or limiting self-care activities of daily living
- Wheezing with severe symptoms limiting self-care activities of daily living, or requiring oxygen therapy or hospitalization
- Dehydration requiring hospitalization
- Hypoglycemia or hyperglycemia requiring hospitalization or long-term changes in medical management
- Hypotension requiring intervention or hospitalization
- Syncope

**Grade 4: Life-threatening**

Life-threatening adverse effects require immediate and urgent intervention to avoid death. Such adverse events include

- Dyspnea (life-threatening) requiring urgent intervention
- Wheezing with life-threatening consequences or requiring urgent intervention
- Dehydration with life-threatening consequences
- Hypoglycemia or hyperglycemia with life-threatening consequences
- Hypotension (life-threatening) requiring urgent intervention
- Cardiac arrest

**Grade 5: Lethal**

Lethal adverse effects lead to death and are generally a continuum of life-threatening events. Such adverse events include

- Dyspnea leading to death
- Wheezing leading to death
- Dehydration leading to death
- Hypoglycemia or hyperglycemia leading to death
- Hypotension leading to death
- Cardiac arrest leading to death

#### Second and Third Trials

The second and third trials of the 6MST will be performed in a randomized order, with either direct (therapist present at the home) or remote (therapist present through videoconferencing software, as in a telerehabilitation setting) monitoring. For the remotely monitored trial, another person (ie, family member, etc) will be present to ensure safety in case of emergency. The 6MST and adverse events will be, respectively, performed and collected as in the first trial (the only difference being the presence of the therapist through videoconferencing for one of the trials). The third trial of the 6MST will be followed by a semistructured interview using a guide created a priori. This interview will focus on the 2 themes, that are (1) level of comfort executing the test (ease of execution, symptoms, and anxiety) and (2) perception of safety (regarding test and home and telerehabilitation settings). The interview will be conducted in person or through videoconferencing software, depending on the randomization of the third trial.

### Data Preparation and Analysis

All data will be stored in digital databases (REDCap [Research Electronic Data Capture] in Québec and OpenClinica in France) on secured servers at the participating research facilities (CR-IUCPQ and GHICL) following local protocols. Using a standardized thesaurus, research teams will enter data directly into both databases through remote access. With regard to data analysis, descriptive statistics will be used to summarize participant characteristics. For quantitative data, normality will be verified with Shapiro-Wilk tests and Q-Q plots. All statistical analyses will be completed using SPSS Statistics Version 28 (IBM) with a significance level set at *P*<.05. Graphical analyses will be conducted using Excel (version 2305; Microsoft Corporation) and qualitative analyses will be performed with Nvivio (version 14; Lumivero).

For objective 1, concurrent validity will be assessed using paired sample *t* tests (or Wilcoxon signed-rank tests) to verify whether peak cardiorespiratory (eg, oxygen consumption, minute ventilation, and heart rate) and symptomology (ie, dyspnea and leg fatigue) values obtained during the 6MST are comparable to the CPET and 6MWT. Pearson correlation coefficients (*r*) will also be calculated to quantify the strength of association between peak cardiorespiratory and symptomology values reached during the 6MST with those reached during the CPET and the 6MWT. Additional Pearson correlation coefficients (*r*) will be calculated to determine the strength of the association between the number of steps taken during the 6MST and peak oxygen consumption and heart rate reached during all 3 tests. The level of correlation will be interpreted as poor (≤0.20), fair (0.21-0.40), moderate (0.41-0.60), good (0.61-0.80), and very good (0.81-1.00) [41]. The agreement will be assessed with Bland-Altman plots and 95% limits of agreement (mean difference ± 1.96 SD of the difference) between peak cardiorespiratory and symptomology values reached during the 6MST and those reached during the CPET and the 6MWT [41].

For objective 2, reliability (trial 2 vs 3) will be assessed using paired sample *t* tests (or Wilcoxon signed-rank tests), Bland-Altman plots (with 95% limits of agreement), and intraclass correlations [42-44]. Intraclass correlations will be interpreted as poor (≤0.50), moderate (0.51-0.75), good (0.76-0.9), and very good (0.91-1.00) [44].

For objective 3, all minor and serious adverse events, for all trials, will be reported. The semistructured interviews will be voice-recorded and transcribed. Thematic content analysis will be used to analyze all verbatim [45]. Briefly, a combination of deductive (based on a priori interview guide) and inductive (based on data) approaches will be used to generate themes and subthemes. Coding will be conducted separately by 2 reviewers, who will compare their results and resolve discrepancies through discussion.

### Ethical Considerations

This research project received ethical approval at the CR-IUCPQ on October 12, 2023 (2024-4075, 22380), and at the GHICL on September 25, 2023 (2023-A01571-44). All participants will provide written free and informed consent before enrollment. All information is provided in French and will be translated into English if needed. All data collected will be stored anonymously (coded numeric ID) on secured servers at participating research facilities. Participants will receive financial compensation in accordance with local standardized fees in participating research centers (value of approximately CAD $40[US $30] per laboratory visit and CAD $10 [US $7] per at-home visit).

## Results

The first participant (study 1) was recruited on February 2, 2024, and will continue for a maximum of 12 months. Recruitment for study 2 is to follow the completion of study 1 and is expected to last for a maximum of 12 months. Data analysis will occur after, and results will be published in peer-reviewed journals.

## Discussion

### Overview

This study aims to provide further evidence regarding the metrological properties of the 6MST in individuals with COPD or ILD. The 6MST is a clinically relevant functional capacity test that is specifically adapted for home-based models of care.

This research project innovates by being among the first to extensively evaluate the metrological properties of the 6MST in a diversified sample of pulmonary lung diseases. Moreover, this study is the first to evaluate the validity and safety of assessing exercise capacity using 6MST through direct and remote monitoring, mimicking home-based and telerehabilitation contexts. Since all the equipment required for the 6MST (ie, stepper) can easily be transported by a therapist, or even shipped to the patient, and requires very minimal space, this test is highly applicable in home-based rehabilitation models of care.

Knowledge derived from this research project will therefore contribute to addressing barriers currently hampering the wider adoption of these new models of care. Ultimately, this research project fits in a continuum of knowledge creation that aligns with the United Nations Sustainable Development Goals by contributing to augmenting health care service delivery (goal 3) and reducing health care access inequalities (goal 11).

### Potential Challenges and Appropriate Mitigation Strategies

Certain potential changes were foreseen and the following mediation strategies will be put into place:

1. Changes in a participant’s health status during the study could introduce bias in the results of repeated measures. To help reduce this risk, repeated testing conducted in the laboratory will be preceded by spirometry to ensure stability in health status. For testing conducted in the home-based settings, signs and symptoms of exacerbations will be collected before each repeated testing.
2. The steppers used in this study use hydraulic pistons to simulate the stepping movements. The resistance provided by the pistons is greater when the fluid inside them is cold and decreases progressively with use as the pistons heat. To avoid inducing a potential bias during repeated tests, a standardized 2-minute warm-up (familiarization) will be conducted followed by a standardized 3 minutes of rest before initiating the test trial. Participants will also be instructed not to use the stepper device other than when instructed to do so during testing.
3. Some participants may have trouble maintaining balance during the 6MST. Participants are allowed to touch a wall or other stable surface to assist in maintaining balance. Nevertheless, for home-based testing in the absence of the therapist (ie, videoconferencing), a third person (eg, family member) will be present to ensure safety. This person will also be instructed on how to react in case of emergency.

### Strengths and Limitations

This research project has strengths and limitations that warrant consideration. With regard to strengths, first, sex parity will be assured and help limit bias, particularly related to the under-representation of people of the female sex in the pulmonary rehabilitation literature [46]. Second, the a priori calculation of sample sizes required will ensure sufficient statistical power to address the study’s objectives. Third, the inclusion of several different and complementary statistical strategies will ensure adequate evaluation of the metrological properties of the included tests. Fourth, the multicenter and multinational nature of this study will improve its external validity and generalizability. Fifth, the inclusion of a mixed methods study design will help ensure that stakeholders (ie, individuals with pulmonary diseases) have input regarding the acceptability and use of the physical tests being validated in this research project. Finally, the publication of this protocol a priori will ensure accountability and any changes made a posteriori will be public knowledge and require justification.

With regard to limitations, first, only individuals with COPD and an ILD will be included in this study. This will limit its generalizability in the population with other pulmonary diseases (eg, asthma and cystic fibrosis). This methodological choice was made in an effort to reconcile feasibility (limited time and budget) and generalizability (ie, heterogeneity). Second, for study 1, participants will be required to perform several physical tests on the same day, potentially leading to fatigue that may alter performances. To address this, tests occurring on the same day are separated by a rest period and are randomized. Moreover, the research team has extensive experience with this type of protocol. Finally, regarding test-retest reliability in study 2, a learning effect is possible as the test will be conducted repeatedly. To limit this effect, the first trial of the test will exclusively serve as a familiarization trial. In addition, a 2-minute warm-up period is allotted before each trial. Nonetheless, as the learning effect may not be completely eliminated, randomization of the second and third trials is performed in order to limit a systemic effect on the test conditions (ie, with a therapist present or through videoconferencing software).

## Appendix

Multimedia Appendix 1 Peer-review report from the Fondation Institut Universitaire de Cardiologie et de Pneumologie de Quebec.

Multimedia Appendix 2 The American Thoracic Society’s recommended standardized instructions and encouragements for the 6-minute walk test that were translated to French in Textbox 3.

Multimedia Appendix 3 The original English instructions proposed for the 6-minute stepper test that were translated to French in Textbox 4.

## Abbreviations

6MST: 6-minute stepper test
6MWT: 6-minute walk test
COPD: chronic obstructive pulmonary disease
CPET: maximal cardiopulmonary exercise testing
CR-IUCPQ: Centre de recherche de l’Institut universitaire de cardiologie et de pneumologie de Québec
GHICL: Groupement des Hôpitaux de l’Institut Catholique de Lille
ILD: interstitial lung disease
SpO_2_: pulsed oxygen saturation

## Data Availability

The data sets generated and analyzed during this study are not publicly available to maintain participant confidentiality but are available from the corresponding author on reasonable request.
