# Supplementary material for: Assessing Functional Capacity in Directly and Remotely Monitored Home-Based Settings in Individuals With Chronic Respiratory Diseases: Protocol for a Multinational Validation Study
Source: JMIR Res Protoc. 2024 Jun 28;13:e57404. doi: 10.2196/57404 (PMC11245655; doi:10.2196/57404)
Supplement: Multimedia Appendix 1 [file resprot_v13i1e57404_app1.pdf]

CENTRE DE RECHERCHE
INSTITUT UNIVERSITAIRE
DE CARDIOLOGIE
ET DE PNEUMOLOGIE
DE QUÉBEC

AFFILIÉ À UNIVERSITÉ LAVAL

FONDATION
INSTITUT UNIVERSITAIRE
DE CARDIOLOGIE
ET DE PNEUMOLOGIE
DE QUÉBEC

François Maltais

**Évaluateur #1**	Cote : 4.4
Résumé: Validation de tests d'effort réalisés à domicile chez des personnes avec des maladies respiratoires chroniques. Objectif 1 : Étudier la validité du test de stepper de 6 minutes (TS6) en le comparant avec des tests reconnus (TDM6 et l'EFx). Objectif 2 : Déterminer la reproductibilité dans un contexte domiciliaire et de téléréadaptation. Objectif 3 : Documenter le profil des effets indésirables et le point de vue de la clientèle lors de la réalisation du TS6 à domicile et en téléréadaptation.<br>Forces: Devis d'étude optimale qui tient compte de la puissance, des comparatifs et comprend une description optimale d'analyses statistiques. Question cliniquement importante en réadaptation. Grande expertise du PI en MPOC et mesures physiologiques associées à l'exercice. Accès à 2 cohortes (Québec, France) adéquates.<br>Faiblesses: L'idée d'utiliser le TS6 n'est pas innovatrice. Malgré sa simplicité et sa disponibilité depuis des années ce test n'a jamais vraiment pris son essor en réadaptation. La suite de cette étude reste donc imprévisible.	

**Évaluateur #2**	Cote: 4.3
Important unmet clinical need. Pragmatic question and appropriate study design. Feasible by this investigator. The need to accurately quantify peak exercise capacity to successfully implement pulmonary rehabilitation could have been better presented.	

**Évaluateur #3**	Cote: 4.0
validation du test de stepper 6 minutes et comparaison avec 6MWT et epreuve d'effort cardiorespiratore maximale. Recrutement MPOC et FP. Quebec et France. Et validation du test a distance (via videoconference). Un tel test peut aider l'evaluation de la capacite fonctionelle a distance, donc utile pour patients qui demeurent en region. faisabilite: oui. surement plus que 30k a realiser. effet levier: modere.	

Score consensus : 4.23
Rang: 8
