# Supplementary material for: Assessing Functional Capacity in Directly and Remotely Monitored Home-Based Settings in Individuals With Chronic Respiratory Diseases: Protocol for a Multinational Validation Study
Source: JMIR Res Protoc. 2024 Jun 28;13:e57404. doi: 10.2196/57404 (PMC11245655; doi:10.2196/57404)
Supplement: Multimedia Appendix 2 [file resprot_v13i1e57404_app2.pdf]

Appendix 2

These are the recommended standardized instructions and encouragements for the 6-minute walk test that were translated to French in textbox 3 [1].

Textbox 6. Direct citation of the American Thoracic Society's recommended standardized instructions and encouragements for the 6-minute walk test [1]

Standardized instructions:

The object of this test is to walk as far as possible for 6 minutes. You will walk back and forth in this hallway. Six minutes is a long time to walk, so you will be exerting yourself. You will probably get out of breath or become exhausted. You are permitted to slow down, to stop, and to rest as necessary. You may lean against the wall while resting, but resume walking as soon as you are able.

You will be walking back and forth around the cones. You should pivot briskly around the cones and continue back the other way without hesitation. Now I'm going to show you. Please watch the way I turn without hesitation. (Demonstration)

Are you ready to do that? I am going to use this counter to keep track of the number of laps you complete. I will click it each time you turn around at this starting line. Remember that the object is to walk AS FAR AS POSSIBLE for 6 minutes, but don't run or jog.

Start now, or whenever you are ready.

Standardized encouragements:

- You are doing well. You have 5 minutes to go.
- Keep up the good work. You have 4 minutes to go.
- You are doing well. You are halfway done.
- Keep up the good work. You have only 2 minutes left.
- You are doing well. You have only 1 minute to go.

Reference

1. American Thoracic Society. ATS statement: guidelines for the six-minute walk test. Am J Respir Crit Care Med. 2002 Jul 1;166(1):111-7. PMID: 12091180. doi: 10.1164/ajrccm.166.1.at1102.
