# Supplementary material for: Assessing Functional Capacity in Directly and Remotely Monitored Home-Based Settings in Individuals With Chronic Respiratory Diseases: Protocol for a Multinational Validation Study
Source: JMIR Res Protoc. 2024 Jun 28;13:e57404. doi: 10.2196/57404 (PMC11245655; doi:10.2196/57404)
Supplement: Multimedia Appendix 3 [file resprot_v13i1e57404_app3.pdf]

Appendix 3

These are the original English instructions proposed for the 6-minute stepper test that were translated to French in textbox 4 [1].

Textbox 7. Direct citation of the original standardized instructions and encouragements for the 6-minute stepper test [1]

**Original standardized instructions:**<br>The object of this test is to make the highest number of strokes you can during a six minute duration. Six minutes is a long time, so you will be exerting yourself. You will probably get out of breath or become exhausted. You are permitted to slow down, to stop, and to rest as necessary. You may lean against the wall while resting, but you have to resume exercise as soon as you are able.<br><br>The correct movement is this one: you have to stretch the bent leg until the step has touched the stepper base. Then do the same movement with the other leg. (Demonstration)

Reference

1. Borel, B, Fabre, C, Saison, S, Bart, F, Grosbois, J-M. An original field evaluation test for chronic obstructive pulmonary disease population: the six-minute stepper test. Clinical Rehabilitation. 2010 2010/01/01;24(1):82-93. doi: 10.1177/0269215509343848.
